# Comparative Analysis of Peptide Composition and Bioactivity of Different Collagen Hydrolysate Batches on Human Osteoarthritic Synoviocytes

**DOI:** 10.1038/s41598-018-36046-3

**Published:** 2018-12-07

**Authors:** Viktor S. Simons, Guenter Lochnit, Jochen Wilhelm, Bernd Ishaque, Markus Rickert, Juergen Steinmeyer

**Affiliations:** 1 Laboratory for Experimental Orthopaedics, Department of Orthopaedics, Justus Liebig University Giessen, Paul-Meimberg-Str. 3, 35392 Giessen, Germany; 2 Protein Analytics, Department of Biochemistry, Faculty of Medicine, Justus Liebig University Giessen, Friedrichstr. 24, 35392 Giessen, Germany; 3 German Lung Research Center, Justus Liebig University Giessen, Gaffkystr. 11, 35392 Giessen, Germany

## Abstract

Collagen hydrolysates (CHs) are heterogeneous mixtures of collagen peptides that are often used as nutraceuticals for osteoarthritis (OA). In this study, we compared the peptide composition and pharmacological effects of three different CH preparations (CH-Alpha^®^, Peptan^®^ B 2000 and Mobiforte^®^) as well as their production batches. Our biochemical analysis using MALDI-TOF mass spectrometry and the ICPL™-isotope labelling method revealed marked differences between different CH preparations and even between some production batches of the same preparation. We also investigated the pharmacological effects of these CHs on human fibroblast-like synoviocytes (FLS). No significant effects on cultured FLS could be demonstrated for either production batch of CH-Alpha^®^, Peptan^®^ B 2000, and Mobiforte^®^ analyzing a small number of pharmacological relevant targets. Thus, our study already shows for the first time that different production batches of the same CH preparation as well as different CH preparations can differ significantly in their peptide composition. In this line, further studies are also needed to verify equal pharmacological efficacy of CH batches on a much broader range of (patho)physiological relevant targets. If OA patients are to be offered a safe and effective nutraceutical a better knowledge about all potential effects as well as ensuring the same active-substance levels are a prerequisite.

## Introduction

Osteoarthritis (OA) is the most common joint disorder affecting approximately 15% of the total^1^ and 60% of the elderly population^2^, in whom it causes pain, restriction of movement and disability^2,3^. During the progress of the disease, OA affects not only the articular cartilage, but also the entire joint including the subchondral bone, synovium, ligaments, and periarticular muscles, and as a low-grade systemic inflammation possibly the entire organism^2–6^. Since it has been shown that OA also has an inflammatory component mainly mediated via the release of soluble inflammatory factors, OA is no longer considered as a degenerative disease caused exclusively by aging and mechanical stress^6^. The fact, that there is no cure for OA, and that treatments are not available for slowing progression down, has led in recent years to an increasing interest both in patients and the scientific community for alternative nutritional treatments that produce reduced side-effects^7–9^. In the US, 47% of patients with OA were reported as using alternative treatments including food supplements and nutraceuticals^9^ such as collagen hydrolysates (CHs).

CHs consist of a mixture of different peptides obtained from collagen-containing tissues by hydrolytic and/or enzymatic degradation^10–13^, and they are generally considered as a safe food ingredient^7^. Since Oesser *et al*.^14^ published an *in vitro* study showing that CHs can stimulate the biosynthesis of collagen by chondrocytes of articular cartilage, CHs have gained a huge amount of public attention. Currently, many different CH preparations are commercially available from various companies who produce those using unpublished manufacturing processes. Collagenous proteins used for the production of CHs are derived from bone and skin, or the scales and fins of various vertebrates^10,12,15^. As such the precise peptide profiles may differ between CH preparations and is often even unknown^11,12,16^.

However, the clinical efficacy of CHs with OA remains unclear: While some studies attested a pain-reducing effect^17,18^, this observation could not or only partly be confirmed in other studies^13,19^. Remarkably, Benito-Ruiz *et al*.^17^ reported a significantly higher pain reduction in patients with less average meat and thus collagen consumption after taking a CH preparation. However, this effect was not found in patients who had high meat consumption indicating that CH could be especially beneficial in patients with low meat intake. In addition, McAlindon *et al*.^19,20^ found some evidence for an anabolic effect on articular cartilage after orally administered collagen peptides in patients using MRI and biomarkers. However, these data are preliminary due to the small sample size, the missing morphometric MRI sequences, and no consistent correlation between PIIANP or CS846 with changes of the dGEMERIC score. It was concluded, that the evidence to recommend the generalized daily use of CHs for patients with OA is not sufficient and no health claim has been approved by the European Food Safety Authority^21,22^. Up until now, alleged effects of CHs have been attributed to the effect of CHs on cartilage tissue. Several studies by different working groups have been performed on cultured animal or human chondrocytes as well as cartilage explants to analyse the effects of CHs and endogenous collagen fragments. However, different and even contradictory results have been obtained in these studies^11,12,14,23–25^.

We already reported that human articular cartilage explants respond differently to various bovine, porcine or fish CH preparations^11,12^. Furthermore, we also found evidence that individual CH preparations induce an elevated release of proinflammatory mediators such as IL-6, PGE_2_, NO and various MMPs^11,12^. Some CHs even inhibited collagen biosynthesis in chondrocytes at high concentrations *in vitro*^11,12^. Using matrix-assisted laser desorption/ionisation (MALDI)-time of flight (TOF)-mass spectrometry (MS), nuclear magnetic resonance spectroscopy and atomic force microscopy, we were able to demonstrate that different CH preparations markedly differ in their biochemical composition^11,12^. As such, the term CH describes a heterogeneous group of non-fibrillar collagenous peptide mixtures and we assume that different effects of CHs are possibly related to variations in the compositions of bioactive peptides^11,12^. However, there is currently no information in the literature as to whether different batches of CH preparations differ with respect to their peptide profiles.

In 2010, Ohara *et al*.^26^ reported that proline-hydroxyproline (Pro-Hyp) peptides, frequently found in sera after oral ingestion of CHs^26–28^, stimulate hyaluronic acid synthesis by rabbit-derived fibroblast-like synoviocytes (FLS)^26^. However, the effects of exogenous and endogenous collagen fragments on synovial membranes or FLS are not currently known. Our study was therefore designed to fill this knowledge gap by systematically investigating the effects of exogenously administered CHs on FLS. Based on the results of our previous investigations^11,12^, the specific aim of our current study was (a) to further quantify the biochemical differences between the peptide profiles of different CH preparations using MALDI-TOF-mass spectrometry, and (b) to test for the first time the reproducibility of the composition of different batches of the same CH preparation. This was further substantiated biologically by determining whether different CH preparations as well as their batches possess bioactive peptides that induce the same cellular effects on FLS obtained from OA-patients.

## Materials and Methods

### Reagents

The bovine CH preparations Peptan^®^ B 2000 (lot no. 1048665 and 1266793/x) and CH-Alpha^®^ (lot no. L115/1031, L88/1031, L170/1031) were from Rousselot (Puteaux, France) and Quiris Healthcare (Gütersloh, Germany), respectively, whereas the porcine CH Mobiforte^®^ (lot no. 11/2016/L07 and 11/2016/L06) was obtained from Astrid Twardy (Unterföhring, Germany). The batches of CH-Alpha^®^ and Mobiforte^®^ contained some additional ingredients such as fructose and vitamin C. Unless otherwise indicated, all reagents were purchased from Sigma (Deisenhofen, Germany). Dulbecco’s modified Eagle media (DMEM) and penicillin/streptomycin were from PAN Biotech (Aidenbach, Germany), SERVA ICPL™ kit was from SERVA (Heidelberg Germany), HEPES was from Invitrogen™ (Karlsruhe Germany), nitrate reductase was from Roche Diagnostics (Mannheim, Germany), and peqGold Trifast™ was from Peqlab Biotechnologie GmbH (Erlangen, Germany).

### MALDI-TOF MS Analysis

As described earlier^12^, the numbers of peaks representing individual peptides and the numbers of common peptides between CH preparations and their batches were estimated by Matrix-assisted laser desorption/ionization (MALDI)-time of flight (TOF)-mass spectrometry (MS) in reflector mode. In addition, the concentrations of peptides in different batches of CH preparations were compared using the Isotope Coded Protein Label (ICPL™) methodology. In order to remove impurities such as additional ingredients, 10 mg/ml of each CH batch were first dissolved in 0.1% trifluoroacetic acid (TFA) and subjected to reverse-phase solid-phase extraction (DSC-18, Supelco, Bellefonte, USA). Bound peptides were washed, eluted with 10 ml 0.1% TFA in 80% acetonitrile, and lyophilised after removal of excess acetonitrile using a speed vac. The samples were then re-solubilised in 1% TFA at a concentration of 10 mg/ml.

For the quantitative comparison of two batches from one CH preparation, each purified batch was labelled with either ^1^H (=ICPL™-0 reagent) or ^2^H (=ICPL™-6 reagent) isotope using the ICPL™ methodology according to the manufacturer’s instructions^29^.

Peptides in unlabelled or ICPL™-labelled samples were subsequently separated by reversed-phase high-performance liquid chromatography (Dionex, Idstein, Germany) on an XBridge™ C18 column (Waters GmbH, Eschborn, Germany) for 1 h resulting in 15 fractions. Fractions were lyophilised and frozen at −20 °C until analysis. The fractionated samples were then re-solubilised in 0.1% TFA and mixed with 2.5-dihydroxybenzoic acid and methylenediphosphonic acid (5 mg/ml each) as the matrix solution. These solutions were then applied to the MALDI-TOF target as 2-µl droplets before being allowed to crystallise. In addition, an internal peptide calibration standard was applied to each MALDI-TOF target. The mass spectra were acquired using a Bruker Ultraflex I TOF/TOF MALDI instrument (Bruker Daltonics, Bremen, Germany) in positive mode with a pulsed nitrogen laser which emitted light at 337 nm. The range of analysis was set from *m/z* 500 to *m/z* 4000 for the reflector mode. The extraction voltage was 25 kV. Each mass spectrum was obtained as an average of approximately 350–750 single laser shots. With the ICPL™-labelled samples, each peptide common to both batches appeared with a defined mass difference Δm of 6.0204 or a multiple thereof in case of multiple peptide labelling. Peptides in both batches were quantitatively compared by calculating the ratio of the two intensities (intensity of the peak labeled with ICPL™-6-reagent/intensity of the peak labeled with ICPL™-0-reagent) obtained for each common peptide labelled with either the ^1^H or ^2^H isotope.

In our quantitative analysis using the ICPL™-method, the *S/N* was set at ≥3. Each batch comparison was measured in triplicate. A peak pair was identified only if the mass difference Δm between the two peaks was 6.0204 or 12.0408 Da to avoid artefacts or interference effects. Furthermore, only peak pairs with a mass deviation of ≤ 50 ppm and which were present within the same fraction of a replicate were taken into account.

When two ICPL™ labelled samples are combined according to the manufacturer’s instructions, there may be a minimal quantitative difference that might distort the results of concentration comparisons. For this reason, we determined a correction factor for each measurement. The correction factor was calculated by determining the arithmetic mean of the intensity ratios of all peak pairs found. The intensities of the peaks that were labeled with the ICPL™-0-reagents were then multiplied by the correction factor. The intensity ratio of each peak pair found was then re-determined using the corrected intensity of the ICPL™-0-labeled peak. Only peak pairs which occurred in all three replicates with a mass deviation of ≤50 ppm and in the same or directly adjacent fraction numbers were used for our quantitative analysis.

In our comparative analysis of different CHs and their batches, the *S/N* was set at ≥10 to avoid any artefact measurements. Each CH was measured in triplicate, and peaks representing individual peptides that were common to all 3 replicate measurements were used to compare the peptide profiles of 2 or 3 CH preparations or batches. The number of detected peaks per 3 replicate measurements of one batch was compared to those of another batch in order to determine whether batches differ with respect to the total number of detected peaks. Only peaks with a mass deviation of ≤50 ppm and which were present in the same or a directly adjacent fraction were considered to be identical.

We further determined various reference values. For this purpose one batch of unlabeled CH-Alpha^®^ (lot no. 170/1031) was analyzed by MALDI-TOF MS on two respectively three different days and the respective percentage of common peaks was determined. For samples labelled with ICPL™ one batch of Mobiforte^®^ (lot no. 11/2016/L07) was labelled with either ICPL™-0 or ICPL™-6 isotopes and the number of peaks with an intensity ratio ≥0.8 and ≤1.2^30^ were determined.

### Specimen Selection for Isolation of FLS

Human synovial tissue containing FLS were obtained from OA knee joints (Kellgren-Lawrence classification: 5x grade 4, 1x grade 3) during knee replacement surgery (n = 6, age 55–79 years, BMI 23.9–31.6 kg/m^2^, CRP 1.8 ± 2.3 mg/ml, both genders, 5 male, 1 female). All procedures performed in the study involving human patients were in accordance with the principles outlined in the Declaration of Helsinki, and approval by the local Ethical Review Committee of the Faculty of Medicine of the Justus Liebig University Giessen had been obtained. All patients provided informed written consent to donate samples for research. All patients had comorbidities which included arterial hypertension (5), coronary artery disease (3), cardiac arrhythmia (2) diverticulosis (2) and asthma (2). OA patients were selected at random from our orthopaedic clinic with the indication for a knee replacement surgery.

### Cell Culture of FLS

Human FLS were isolated from synovial membranes as described elsewhere^31^. FLS were cultured in a humidified 10% CO_2_ atmosphere at 37 °C using DMEM medium supplemented with 1.0 g/l glucose and 584 mg/l L-glutamine, 10% foetal bovine serum (FBS), 10 mM HEPES buffer, 10 U/ml penicillin and 0.1 mg/ml streptomycin. The experiments were performed with cells harvested at the end of passages 3 to 5. All cells tested negative for mycoplasma contamination using the PCR mycoplasma test kit I/C (PromoCell, Heidelberg, Germany). The purity of our FLS was ensured by means of fluorescence activated cell sorting (FACS) using monoclonal mouse anti-human fibroblast surface protein antibody SM1214PS (clone D7-FIB, Acris Antibodies, Herford, Germany) as a positive control. Positive and negative controls were carried out using an anti-HLA-DR-antibody and a polyclonal rabbit anti-mouse immunoglobulin conjugated with R-phytoerythrin (code no. R 0439, Dako, Glostrup, Denmark). 89% ± 4% of the cultured cells were stained positively for fibroblast-specific antigen.

In our experiment, FLS (approx. 80.000 in each sample) obtained from 6 different patients were cultured for 48 h in the presence of 0–5 mg/ml CH. CHs were dissolved in 4 mL DMEM with 2% FBS which was also used as the vehicle for untreated controls. Cells were serum-starved to 2% FBS for 24 h prior to treatment lasting 48 h. Then, the cellular protein content and the levels of different enzymes and cytokines within the nutrient media were determined. Cells were lysed using peqGold Trifast™ according to the manufacturer’s instruction, before being frozen at −86 °C until further analysis. Nutrient media were frozen at −20 °C in the presence of 10% (vol/vol) protease inhibitor cocktail cOmplete™ (Roche Diagnostics, Mannheim, Germany) until further analysis.

### Determination of Cellular Protein Content

To normalise the amount of cytokines and enzymes analysed in culture media, the cellular protein content of FLS was determined in triplicate using the bicichoninic acid assay (Pierce™ BCA Protein Assay, Thermo Fischer Scientific, Dreieich, Germany) according to the instructions provided by the manufacturer.

### Determination of MMP-1, MMP-13, TIMP-3 and NO

MMP-1, MMP-13 and TIMP-3 levels in the culture media of FLS were measured using commercially available MMP-1, MMP-13 and TIMP-3 ELISA kits from RayBiotech (Norcross, USA) and R&D Systems (Wiesbaden, Germany) respectively, according to the manufacturer’s instructions. Data were normalised with respect to the cellular protein content. NO production of FLS was measured in the culture media in duplicate using the Griess reaction after reduction of the nitrate by nitrate reductase. Sodium nitrite was used as a standard as previously described in more detail^12,32,33^.

### Determination of IL-1ß, IL-6, IL-8, PGE_2_ and TNF-α

IL-1ß, IL-6, IL-8 and PGE_2_ were measured in the FLS culture media using commercially available ELISA kits (IL-1ß Quantikine® ELISA from R&D Systems, Wiesbaden, Germany, IL-6 High Sensitivity Kit from eBioscience, San Diego, USA; Human CXCL8/IL-8 Immunoassay from R&D Systems, Wiesbaden, Germany; PGE_2_ Kit from Cayman Chemical Company, Ann Arbor, USA) according to the manufacturer’s instructions. TNF-α was quantified using two ELISA kits with different sensitivities (Human TNF-α kit from R&D Systems, Wiesbaden, Germany and Human TNF-α UltraSensitive Kit from Invitrogen™, Karlsruhe, Germany). Cultured medium to be analysed was concentrated fivefold using a centrifugal filter (Amicon^®^ Ultra-0.5 ml 3K-device from Merck, Darmstadt, Germany) to allow small amounts of TNF-α to be detected. All data were normalised with respect to the cellular protein content.

### Statistical Analysis of Data

**Figure 1:**
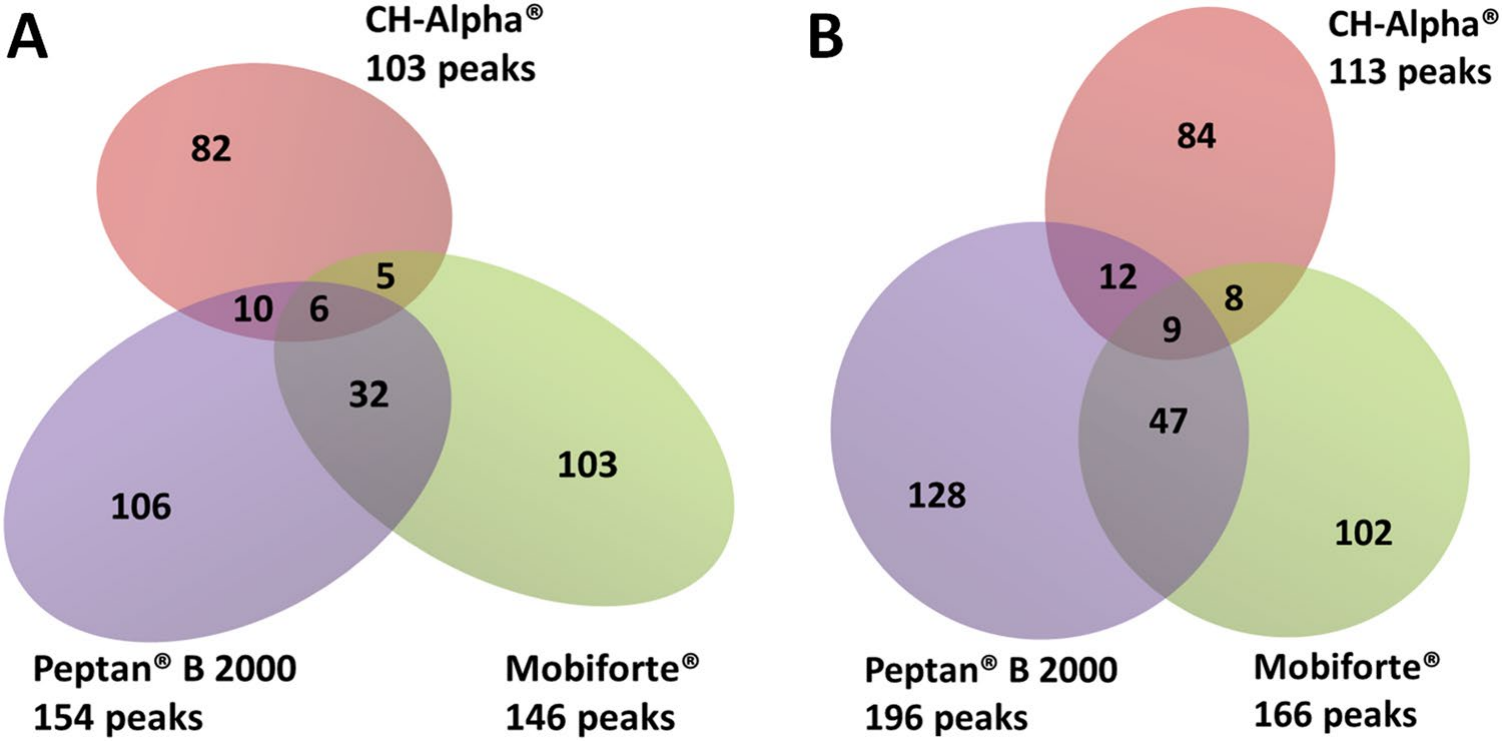
Number of peptides in different batches of Mobiforte^®^, CH-Alpha^®^ and Peptan^®^ B 2000 as determined by MALDI-TOF-MS analysis. The numbers outside the diagram represent the number of total common peaks obtained from 3 replicate measurements, with each peak representing a peptide of the corresponding batch. The numbers in the intersections of the Venn diagrams are peptides shared by one, two or by all preparations. Analysed batches: (**A**) CH-Alpha^®^ (lot no. L115/1031), Peptan^®^ B 2000 (lot no. 1048665) and Mobiforte^®^ (lot no. 11/2016/L07); (**B**) CH-Alpha^®^ (lot no. L88/1031), Peptan^®^ B 2000 (lot no. 1266793/x) and Mobiforte^®^ (lot no. 11/2016/L06).

**Figure 2:**
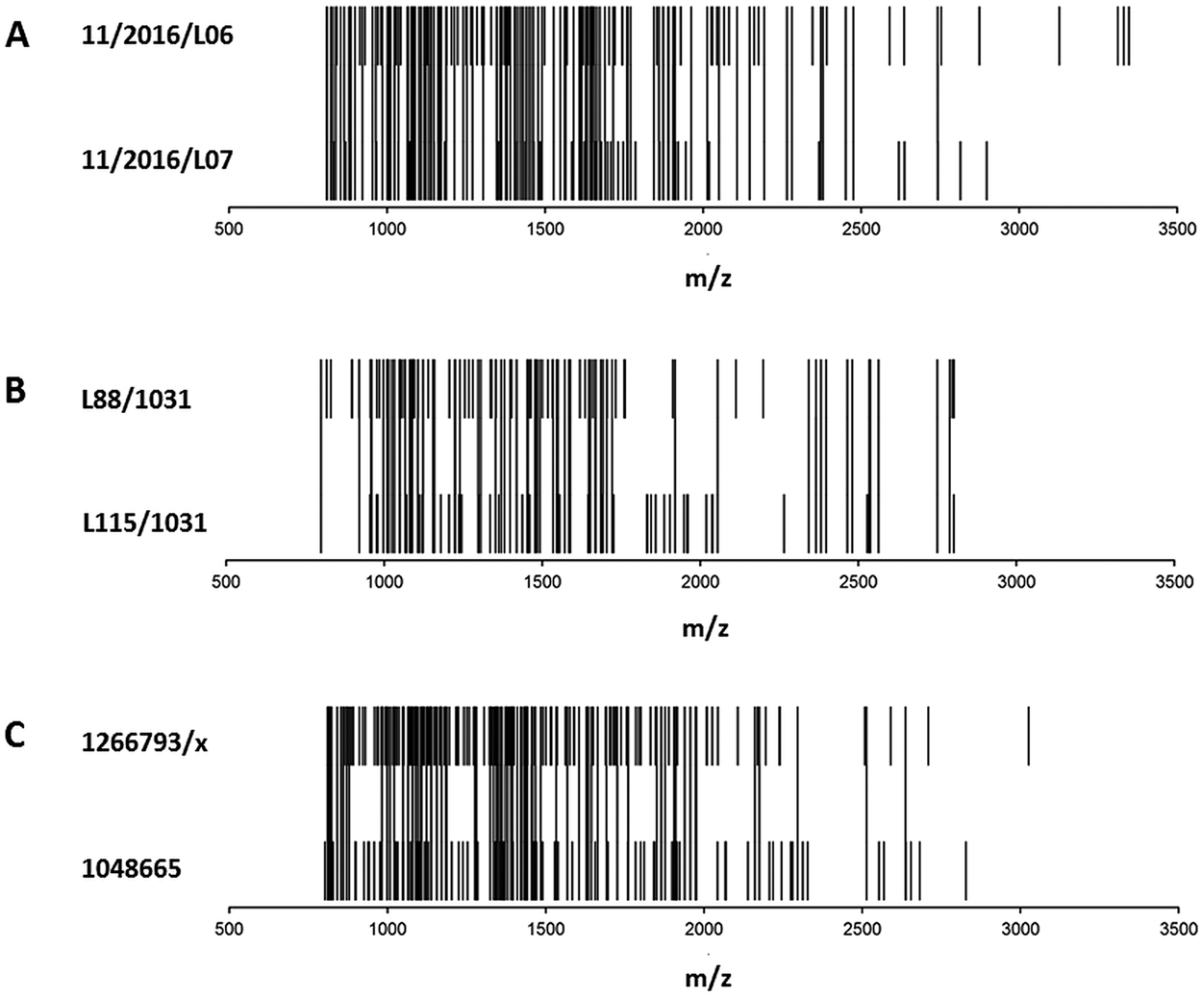
Comparative analysis of MALDI-TOF mass spectra obtained from two batches of (**A**) Mobiforte^®^, (**B**) CH-Alpha^®^, and (**C**) Peptan^®^ B 2000. Each peak of the rug plots represents a common peptide obtained from 3 replicate measurements in one batch. If a common peptide of one batch was also found in the other batch, it is marked as a continuous peak.

**Figure 3:**
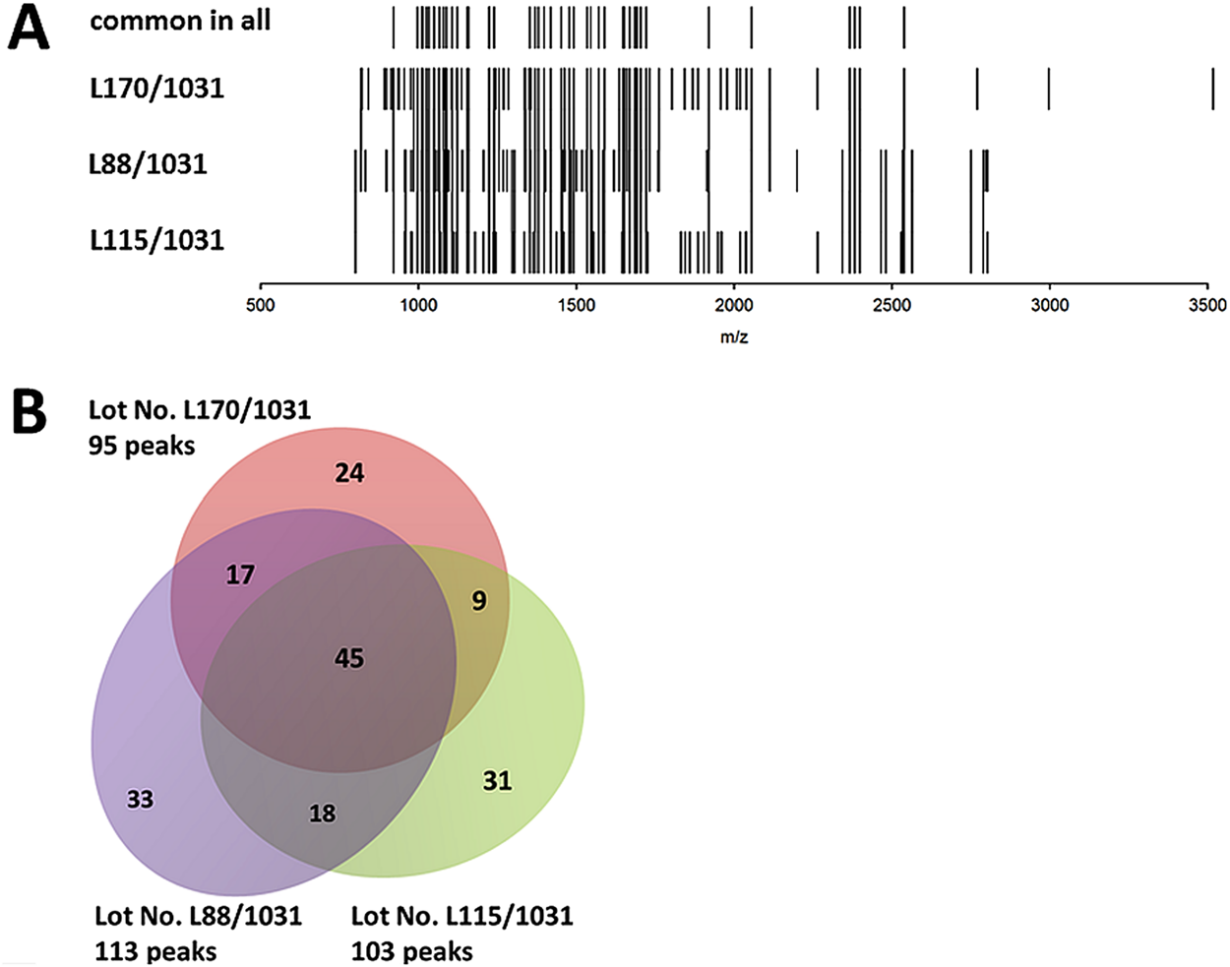
Comparative analysis of MALDI-TOF mass spectra obtained from three different CH-Alpha^®^ batches. The mass spectra of the batches with lot no. L115/1031, L88/1031 and L170/1031 are presented as (**A**) a rug plot, and (**B**) a Venn diagram. Each peak of the rug plot represents a common peptide obtained from 3 replicate measurements of one batch. If a common peptide of one batch was found in two or three batches, it is marked as a continuous peak. The numbers outside the Venn diagram are the number of total peptides present in each batch. The numbers in the intersections are peptides shared by one, two or by all batches.

Each CH preparation was analysed by MALDI-TOF-MS in three independent measurements (n = 3). Cell culture experiments were repeated 5–6 times using FLS each time obtained from different patients (n = 5–6). In order to determine whether different CH preparations or batches differ with regard to their peptide profiles, the percentages of common peaks found in two or three CH preparations (Fig. 1, Suppl. Table 1) or batches (Figs 2 and 3, Suppl. Table 2) were compared with the respective values from our reference measurements using Fisher’s exact test. The number of peptides in two different CH batches or preparations was compared using an unpaired, two-tailed t-test. Reproducibility of our MS analysis was determined by comparing the percentage of reproducibly detected peaks from each CH preparation and batch using an unpaired, two-tailed t-Test (Table 1). The significance threshold was set to p ≤0.05.

**Table 1:** Total number of peaks detected as well as percentage of reproducible peaks in each of the three replicated measurements of different CH preparations using a S/N of ≥ 10.

CH-preparation	Lot No.	Total number of peaks per replicate (mean ± SD)	Percentage of reproducible peaks per replicate (mean ± SD)
Peptan^®^ B 2000	1048665	365/266/243 (291 ± 65)	46.3/58.3/63.8 (56 ± 9)
	1266793/x	324/277/319 (307 ± 26)	60.5/70.8/61.4 (64 ± 6)
Mobiforte^®^	11/2016/L07	245/288/320 (284 ± 38)	59.6/50.7/45.5 (52 ± 7)
	11/2016/L06	282/221/268 (257 ± 32)	58.9/ 75.1/61.9 (65 ± 9)
CH-Alpha^®^	L115/1031	170/174/165 (170 ± 5)	60.6/59.2/62.4 (61 ± 2)
	L88/1031	164/212/182 (206 ± 40)	68.9/ 53.3/62.1 (61 ± 8)
	L170/1031	172/171/151 (165 ± 12)	55.8/56.1/63.6 (59 ± 4)

**Figure 4:**
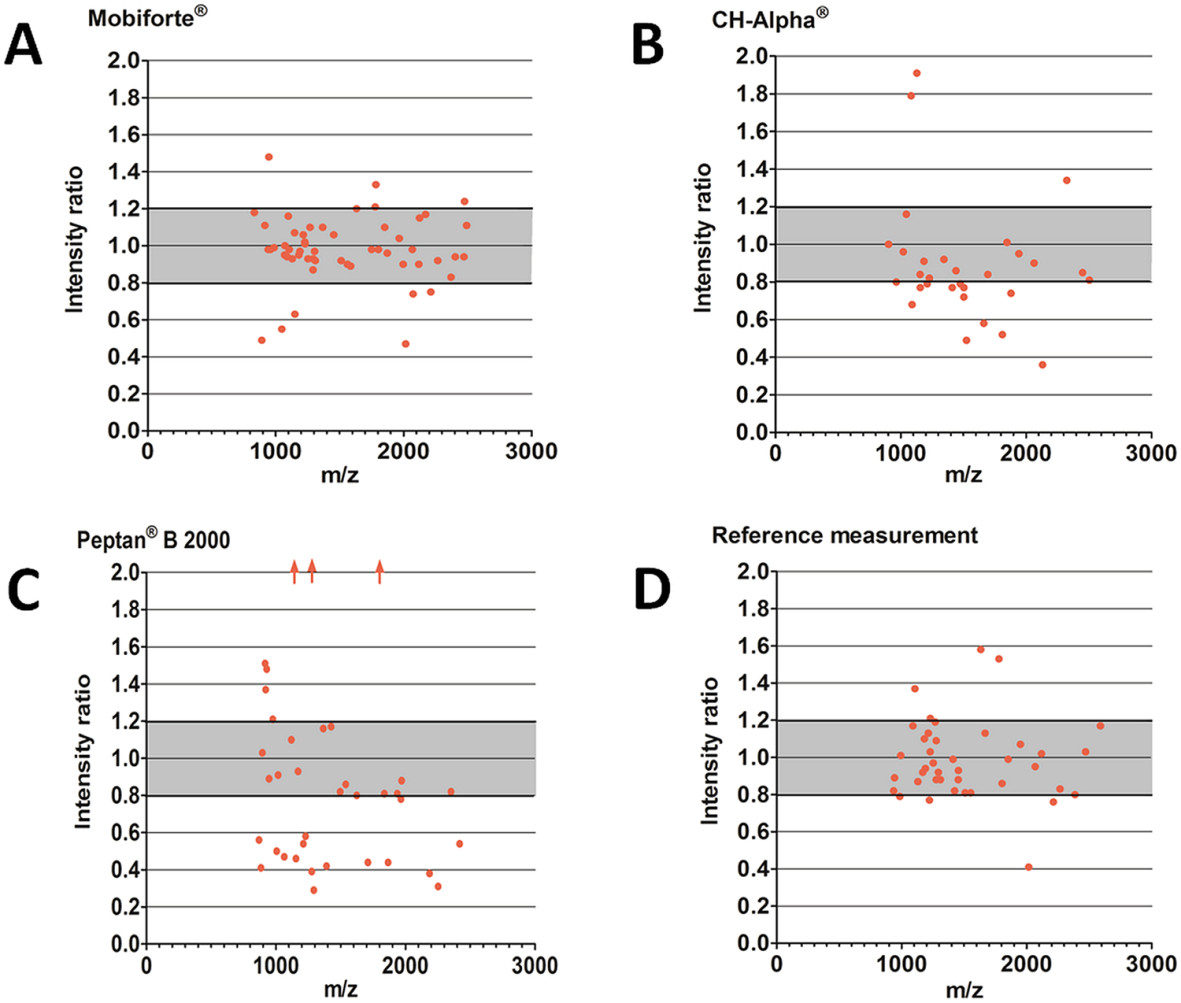
The relative abundance of peptides in different batches of (**A**) Mobiforte^®^ (lot no. 11/2016/L06 and 11/2016/L07), (**B**) CH-Alpha^®^ (lot no. L115/1031 and L88/1031), (**C**) Peptan^®^ B 2000 (lot no. 1048665 and 1266793/x), and (**D**) reference measurement using Mobiforte^®^ (lot no. 11/2016/L07) labelled with both ICPL™-0 and ICPL™-6 isotopes. To compare the concentrations of peptides, the ratios of peak intensities for each peak pair were determined using the ICPL™-labelling method in the replicates. The mean ratios of peak intensities found in each batch are presented (n = 2–3). Peak pairs with an intensity ratio ≥0.8 and ≤1.2 were considered to represent equal intensities of labelled peak pairs and as such equal concentrations of the same peptide in both batches^30^.  = mean ratio of peak pair >2.

In order to check whether two different batches of CHs differed regarding their peptide profiles (Fig. 4, Suppl. Table 2), the number of peak pairs with a mean intensity ratio ≥0.8 and ≤1.2^30^ was determined and compared with the respective number from our reference measurement using Fisher’s exact test (p ≤0.05). The average intensity ratio of a peak pair was determined as the mean of the intensity ratios of the respective three replicates. Outliers in the three replicates (1–6 per batch comparison) were not biochemically-physically explainable and were excluded by Dean-and-Dixon-outlier-test.

The two-tailed Wilcoxon matched-paired signed rank test was calculated to examine whether different CH preparations showed an effect on the levels of cytokines and enzymes within media of cultured FLS. Obtained p-values were adjusted for multiple comparison according to Holm. The significance threshold was set at p ≤0.05. All data presented within the text are means ± SD. Evaluation of the mass spectrometric analyses was performed using flexAnalysis 3.4 (Bruker Daltonik, Bremen Germany). Statistical analysis and graphics were created using Prism^®^ 5.03 (GraphPad Software Inc., La Jolla, CA, USA).

## Results

### MALDI-TOF-MS Analysis of CHs

MALDI-TOF-MS, performed in reflector mode, covering mass-to-charge ratios (*m/z*) of between 500 and 4000, showed remarkable differences between the peptide profiles of differing CH preparations and between those of some CH-batches. As shown in Fig. 1, the batches of Mobiforte^®^, CH-Alpha^®^ and Peptan^®^ B 2000 differed between each other with respect to the total number of reproducible peaks representing collagenous peptides. We determined significantly less peptides in two batches of CH-Alpha^®^ (103, 113 peaks) than was the case in two batches of Mobiforte^®^ (146, 166 peaks) and of Peptan^®^ B 2000 (154, 196 peaks).

The batches of the three different CH preparations also differed with regard to their peptide composition. Figure 1 demonstrates that only a few common peptides were found in any two or even all CH preparations. Illustrating this fact, the three CH preparations shown in Fig. 1 had only 6 or 9 peptides in common, depending on which batches were being compared. CH-alpha^®^ and Mobiforte^®^ shared 11 or 17 peptides and the two bovine CH preparations CH-Alpha^®^ and Peptan^®^ B 2000 had 16 or 21 peptides in common, depending on which batches were being compared. The percentage of peaks common to all three CH preparations shown in Fig. 1 was 2%, which was significantly less than our reference value of 42% (p < 0.0001). The reference value was obtained by determining the percentage of common peaks in one CH preparation (CH-Alpha^®^ lot no. L170/1031) over three consecutive days (Suppl. Fig. 1). Thus a value for the reproducibility of repeated MALDI-TOF measurements was obtained.

Furthermore, we also compared two or three batches of Mobiforte^®^, CH-Alpha^®^ and Peptan^®^ B 2000 and observed that some CHs have batches showing markedly different peptide compositions (Figs 2 and 3). The two Mobiforte^®^ batches revealed 109 peaks (54%) in both preparations, whereas the two CH-Alpha^®^ batches only revealed 63 common peaks (41%). However, only 45 common peaks (25%) were detected where three different CH-Alpha^®^ batches (Fig. 3) were compared with one another. Figure 2C illustrates that the two Peptan^®^ B 2000 batches revealed 83 peaks (31%) in common. Our reference measurements (Suppl. Fig. 1) showed that only 69 (51%) and 60 (42%) of the peaks were common to two or three independent measurements of the same CH-Alpha^®^ batch (lot no. L170/1031). As such, the two batches of Peptan B 2000 (p = 0.006) and the three different batches of CH-Alpha^®^ (p = 0.016) differed with respect to the percentage of peaks common to all batches. Compared with the reference measurements (51%), the percentages of common peaks between two batches of CH-Alpha^®^ (41%) and Mobiforte^®^ (54%) were similar. A list of the analysed peaks determined in the different CH batches is shown in Suppl. Tables 1 and 2. In none of the batch comparisons did the number of detected peaks differ significantly from one another (CH-Alpha^®^ p = 0.32, Mobiforte^®^ p = 0.40 and Peptan^®^ B 2000 p = 0.44) (Table 1).

We determined whether MALDI-TOF-MS delivers reproducible numbers of peaks at a signal-to-noise ratio (S/N) of ≥10. The reproducibility was quantified by counting the number of peaks in triplicate for each CH preparation or batch and calculating the percentage of reproducible peaks found. The mean percentage of reproducible peaks was 60%, and revealed no significant differences in any of the investigated CH preparations or batches (Table 1).

### Quantitative MALDI-TOF-MS Analysis of CHs with the ICPL™ Labelling Method

The isotope coded protein label (ICPL™) technique was applied to compare the concentrations of peptides in different batches of Mobiforte^®^ (lot no. 11/2016/L07 and 11/2016/L06), CH-Alpha^®^ (lot no. L115/1031 and L88/1031), and Peptan^®^ B 2000 (lot no.1048665 and 1266793/x) by MALDI-TOF-MS (Fig. 4). The ratios of peak intensities of each pair of differently isotope-labelled peaks were determined, and ratios lying in the range of 1.0 ± 0.2 were considered to represent equal intensities of labelled peak pairs and as such equal concentrations of the same peptide in both batches^30^. Figure 4 shows that in some cases there are markedly different concentrations of peptides analysed in different batches of the same CH preparation.

The percentage of peak pairs considered as equivalent from the two batches of Mobiforte^®^ was 78% (Fig. 4A, Suppl. Table 1), whereas these figures for CH-Alpha^®^ and Peptan^®^ B 2000 were only 47% and 34%, respectively (Fig. 4B,C; Suppl. Table 1). Our reference measurement as shown in Fig. 4D revealed that 78% of all determined ratios of peak intensities lay within the limits of between 0.8 and 1.2. As such the batch comparisons for the CH-Alpha^®^ and Peptan^®^ B 2000 batches revealed that these preparations were significantly more inconsistent compared to the comparison of the reference measurements (with p values of 0.012 and ≤0.001, respectively). Remarkably, compared with our reference value of 78%, no statistically significant inconsistency was observed between the two Mobiforte^®^ batches analysed.

### The Collagen Hydrolysates and the Levels of MMPs, cytokines, PGE_2_ and NO

Since we previously reported that CH preparations differ with respect to their potential to modulate the metabolism of human articular cartilage explants^11,12^, we then investigated batch specific biological effects on cultured human FLS. Our analysis revealed that none of the CH preparations or batches showed any statistically significant activity regarding the levels of MMP-13, TIMP-3 IL-1ß, IL6, TNF-α, PGE_2_ or NO, even though a broad range of concentrations between 0.1 and 5 mg/ml was tested (data not shown).

### IL-8 and MMP-1 after Treatment with Different Batches of CHs

**Figure 5:**
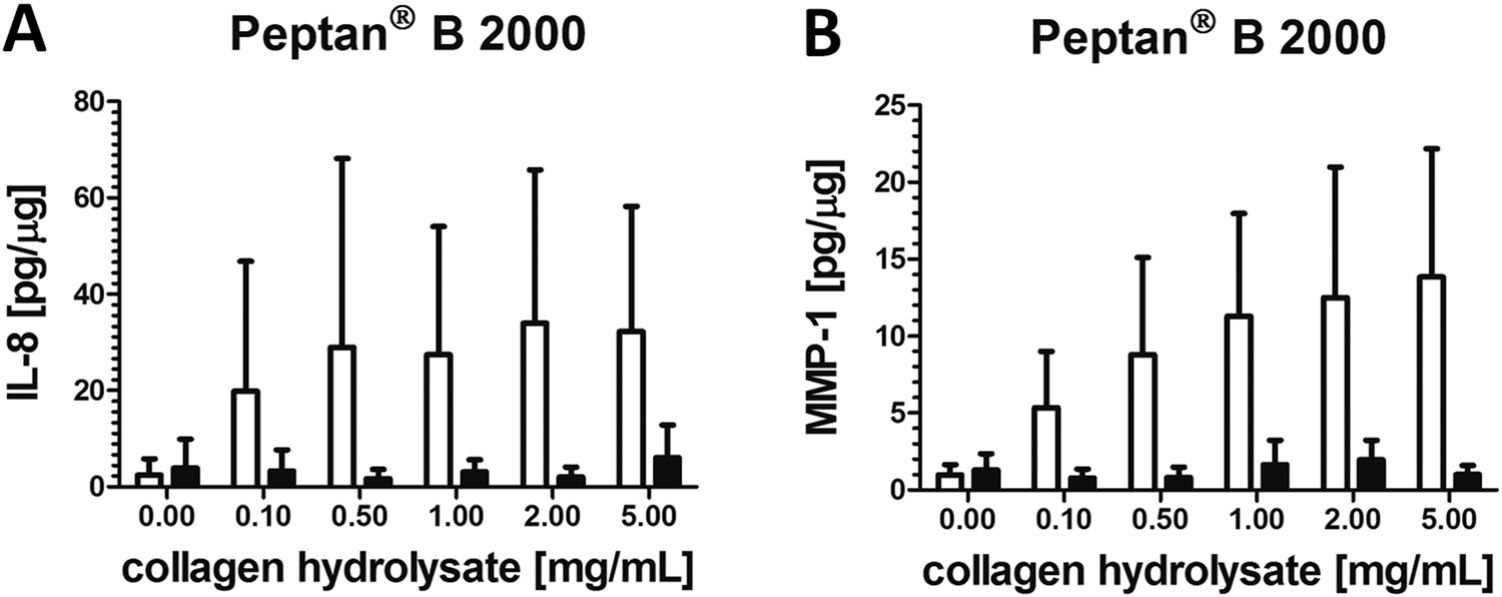
The effects of two different Peptan^®^ B 2000 batches on (**A**) IL-8 and (**B**) MMP-1 content in culture media expressed as pg per µg cellular protein content. IL-8 and MMP-1 were quantified by ELISA in the media of cultured fibroblast-like synoviocytes treated with 0–5 mg/ml Peptan^®^ B 2000. Data are expressed as means ± SD (n = 4–6). □ = Peptan^®^ B 2000, lot no. 1048665; ■ = Peptan^®^ B 2000, lot no. 1266793/x.

Figure 5 demonstrates that only Peptan^®^ B 2000 considerably stimulated the release of IL-8 and MMP-1 into the culture media of synoviocytes although no statistical significant effect could be found. Figure 5 also reveals that only one batch of Peptan^®^ B 2000 (lot no. 1048665) was able to display these effects on cultured human FLS.

## Discussion

The application of nutraceuticals including CHs is becoming increasingly popular even though there is only limited information regarding their clinical efficacy, mode of action or safety^22^. We previously reported that several CH preparations obtained from different sources can vary markedly with respect to both their effects on human articular cartilage as well as their biochemical composition^11,12^. Based on these results, the aim of our current study was to deepen our insights into the natural product CH by comparing both the peptide composition as well as the bioactivity of individually produced batches of various CH preparations. Our novel findings are that (a) the peptide composition and relative abundance of specific peptides can differ between two batches of the same CH preparation, and that (b) the altered peptide composition of the two production batches did not lead to inconsistent bioactivity towards cultured FLS using a limited number of pharmacological targets.

Using MALDI-TOF-MS, we found that the peptide composition differed significantly between the three bovine or porcine CH preparations, namely Peptan^®^ B 2000, CH-Alpha^®^ and Mobiforte^®^. The two batches obtained from the bovine Peptan^®^ B 2000 and those from the porcine Mobiforte^®^ differed significantly from the two bovine CH-Alpha^®^ batches regarding the total number of peaks representing peptides as well as their individual peptide compositions. Only 2% of all peptides were shared by all three CH preparations, whereas the two bovine preparations shared 15% or 18% peptides, depending on the batches analysed. Our data indicate that both the source as well as the production method may have a lasting effect on the peptide composition of CHs.

Furthermore, we report here for the first time that even two batches of the same CH preparation can sometimes display markedly altered peptide profiles. We determined that only 31% of the peaks were common to two batches of Peptan^®^ B 2000, which was significantly less than was the case with our reference measurement (51%). Similar levels of peak consistency were found between both batches of Mobiforte^®^ (54%) and CH-Alpha^®^ (41%). However, when we compared three different batches of CH-Alpha^®^, significantly fewer numbers of common peaks (25%) were found compared to the corresponding reference measurements (42%). Remarkably, the total number of peptides did not differ between the three batches of CH-Alpha^®^. Thus, as a natural product, some inherent alteration in peptide composition may occur, but it should nevertheless be possible to avoid excessive batch inhomogeneity so that more reproducible compositions of CH batches can be achieved.

By applying the ICPL™-methodology, we also investigated whether two batches of Mobiforte^®^, CH-Alpha^®^ or Peptan^®^ B 2000 differ with respect to the relative abundance of their common peptides. We found that only the two tested batches of Mobiforte^®^ were produced in a quantitatively reproducible manner showing equivalent amounts of the most common peptides. This was not observed in the batch comparisons for CH-Alpha^®^ or Peptan^®^ B 2000, where major inconsistencies were revealed.

Taken together, our comparative chemical analysis of the peptide profiles of two batches revealed that reasonable reproducibility could only be seen for the Mobiforte^®^ batches. The CH-Alpha^®^ batches differed mainly due to altered concentrations of peptides, whereas the two Peptan^®^ B 2000 batches showed discrepancies regarding both the actual composition and abundance of the peptides.

For unlabelled samples, a *S/N* of ≥10 was chosen in order to avoid any artefact effects. A *S/N* of ≥3 was chosen for the ICPL™-labelled samples in order to increase the sensitivity since this labelling method provides an additional possibility for peptide identification by determining peak pairs. A correction factor was determined for each measurement to correct for minimal quantitative differences between two combined ICPL™-labelled samples. Each CH was measured in independent replicates (n = 3), and only common peaks between all three replicate measurements were considered as peptides. All measurements were performed in the precise reflector mode by MALDI-TOF-MS. The mass range in our analysis was set from *m/z* 500 to 4000. Thus, peptides with higher mass numbers were not detected by our measurements. However due to their mean molecular mass of 2 to 6 kDa^10,13^, the measured mass range was sufficient to allow general statements about CH preparations to be made.

Reproducibility of measurement is still a major challenge in MALDI-TOF-MS. We therefore tested the reproducibility of our MS-method by investigating whether the percentage of reproducible peaks, as determined in triplicate for each preparation/batch, remained the same at a *S/N* of ≥10. The mean percentage of reproducible peaks was 60% and did not significantly change in any of the investigated CHs, so that different CH preparations and batches could be considered as comparable. We also determined various reference values. For this purpose, one batch of unlabelled CH-Alpha^®^ (lot no. L170/1031) was analysed by MALDI-TOF MS on two and three different days. Only 51% or 42% of the peaks were found to be common after two or three days, respectively. For samples labelled with ICPL™, one batch of Mobiforte^®^ (lot no. 11/2016/L07) was labelled once with ICPL™-0 and once with ICPL™-6 isotopes. In this reference measurement, only 78% of all determined peptide concentrations were found to be equivalent. Our reference values are markedly below the theoretically achievable 100%, and thus reflect the limited reproducibility of the MALDI-TOF-MS analyses.

When comparing the number of common peaks found in both batches of CH preparations using the ICPL™-isotope labelling method, fewer peptides were found than was the case with the unlabelled samples. We only counted those labelled peptide pairs which had a mass difference of 6.0204 or 12.0409 to avoid any effects of interference and possible false-positive results, although peptide pairs with greater mass differences were theoretically possible. We also assume that it was only those peptides that were present at higher amounts within the CH-batches that might have been labelled adequately with the ICPL™ reagents to allow a subsequent detection.

Since different CHs can stimulate a catabolic reaction in human cartilage explants^11,12^, we investigated whether the different CHs had an effect on cultured FLS obtained from human OA knee joints. Our study indicates that two batches of the same CH preparation have indeed no significant different effect on FLS using a limited number of pathophysiological relevant targets.

A daily dose of 10 g CH is recommended by some manufacturers, so that the serum levels of CH are most likely below 2 mg/ml and probably even below 1 mg/ml due to the (assumed) restricted bioavailability^11,12^. Thus in our experiments, we included physiologically achievable concentrations which were also similar to those used in most other *in vitro* studies carried out using chondrocytes^11,12,14,25,34^. However, in our study, a markedly elevated release of proinflammatory mediators and enzymes could often only be detected at CH concentrations equal or above 1–2 mg/ml, i.e. levels which are probably not achievable in synovial fluid *in vivo*. Furthermore, CHs administered *in vivo* are subjected to further metabolism during resorption and circulation so that only a small amount of orally given peptides will ultimately reach the joints without being modified^12^.

Only one batch of Peptan^®^ B 2000 showed an increased release of MMP-1 into culture media which was non-significant probably due to the small number of replicates. An increased release of various MMPs, such as MMP-1, is a characteristic feature of synovial inflammation during OA which contributes to tissue damage and an inflammatory response^5,35–37^. We previously reported about the effects of Mobiforte^®^, CH-Alpha^®^ and Peptan^®^ B 2000 on cartilage explants^11,12^ using other batches. Similar to our current study, a number of CHs displayed a few proinflammatory effects on cartilage explants. As one example, when cartilage explants were incubated with Mobiforte^®^, increased levels of NO, IL-6 and MMPs were found in the culture medium^12^, whereas in this study neither batch of Mobiforte^®^ displayed an effect on FLS. We assume that these differences may be due to batch specific and/or cell type specific effects. Also, our previous studies^11,12^ revealed that none of the preparations even when used at high concentrations of 10 mg/ml were cytotoxic to chondrocytes of the superficial, intermediate and radial zones of explanted cartilage.

## Conclusion

This study clearly shows that two batches of the same CH preparation may differ significantly with regard to their biochemical composition. However, further studies are needed to verify whether different CH batches possess equal pharmacological efficacy by analyzing a much broader range of physiological relevant targets. We conclude that both the source and the production process ultimately determine the composition and reproducibility of the various CH batches. Based on our findings, it is doubtful whether general statements on the effectiveness, safety, and mode of action of CH preparations and even different batches of CH preparations can be made. As such, our results only serve to reinforce the assumption that the biological effects of CHs observed by us and others are brought about only by a small number of the individual bioactive peptides^11,12,28^. Although the results of our study can only be transferred with considerable reservations to the much more complex situation seen *in vivo*, they suggest a thorough and broad examination of each and every CH preparation with regard to their ability to reproducibly induce various effects both *in vitro* and *in vivo* so that they can be attested as safe and effective nutraceuticals for OA patients.

## Electronic supplementary material

Supplementary Information
