# Supplementary material for: Comparative Analysis of Peptide Composition and Bioactivity of Different Collagen Hydrolysate Batches on Human Osteoarthritic Synoviocytes
Source: Sci Rep. 2018 Dec 7;8:17733. doi: 10.1038/s41598-018-36046-3 (PMC6286367; doi:10.1038/s41598-018-36046-3)
Supplement: Supplementary file 1 — Supplementary Information [file 41598_2018_36046_MOESM1_ESM.pdf]

Supplementary Information

Comparative Analysis of Peptide Composition and Bioactivity of Different Collagen Hydrolysate Batches on Human Osteoarthritic Synoviocytes

by

Viktor S. Simons[1], Guenter Lochnit[2], Jochen Wilhelm[3], Bernd Ishaque[1], Markus Rickert[1] and Juergen Steinmeyer[1,*]

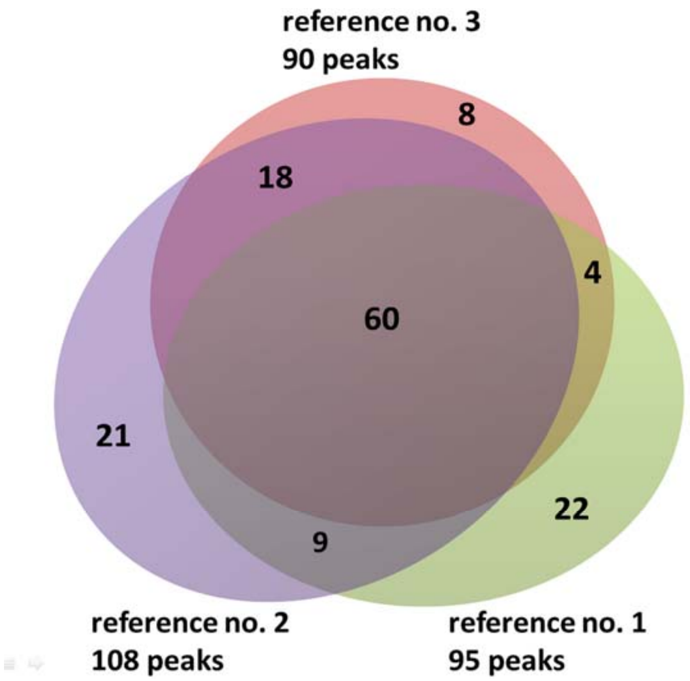

Suppl Figure 1. Common peaks of CH-Alpha® (lot no. L170/1031) used as reference values for comparing collagen hydrolysate preparations and batches. Peaks representing peptides and peptide fragments were determined by MALDI-TOF MS analysis of one CH-Alpha® batch on three different days. The numbers shown outside the diagram are the numbers of total peaks obtained on each of the three days. The numbers in the intersections are peptides shared by one, two or by all three reference measurements. The percentage number of common peptides obtained in the reference measurements no. 1 and no. 2 was 51% (= 69 peaks) and was used as the reference %-value for comparison of two collagen hydrolysate preparations or batches. The percentage number of common peptides present in all three reference measurements was 42% (= 60 peaks) and was used as the reference %-value to compare three preparations or batches of collagen hydrolysates.

**Comparison of product batches**	**Total number of peak pairs**	**Number of peak pairs with an intensity ratio ≥ 0.8 and ≤ 1.2**	**Comparison with reference measurement (p-value)**
**Mobiforte®** (lot no. 11/2016/L07 and 11/2016/L06)	55	43 (= 78%)	≥ 0.05
**CH-Alpha®** (lot no. L115/1031 and L88/1031)	30	14 (= 47%)	0.012
**Peptan® B 2000** (lot no. 1048665 and 1266793/x)	38	13 (= 34%)	≤ 0.001

Suppl. Table 1. Number of peak pairs in the batch comparisons of three different collagen hydrolysate preparations as determined using the ICPL™-labelling method. The number of peak pairs with an intensity ratio ≥ 0.8 and ≤ 1.2 were considered to represent equal intensities of labelled peak pairs and as such equal concentrations of the same peptide in both batches[49] and were compared using Fisher's exact test with the corresponding number of our reference measurement, which was 31 (= 78%). For our reference measurement, a batch of Mobiforte® (lot no. 11/2016/L07) was double-labelled with both ICPL™-0 and ICPL™-6 isotopes. Then the ratios of peak intensities of each peak pair were determined.

Mobiforte® lot no.	
**11/2016/L07**	**11/2016/L06**
**Number of peaks: 146**	**Number of peaks: 166**
**809**	**809**
**810**	**810**
**821**	**821**
**828**	**828**
830	**837**
**837**	841
**853**	**853**
**864**	**864**
869	**879**
**879**	**882**
**882**	**886**
**886**	**898**
**898**	914
**922**	**922**
**954**	930
**964**	**954**
966	**964**
**985**	971
**1001***	980
**1001***	**985**
**1005**	**1001***
**1008**	**1002***
**1011**	**1005**
**1023**	**1008**
**1024**	**1011**
1035	**1023**
**1036**	**1024**
**1064**	1026
**1067**	1029
1071	**1036**
**1076**	1043
**1080**	**1064**
**1085**	**1067**
**1089**	**1076**
**1101**	1079
**1108**	**1080**
**1117**	**1085**
**1123**	1085
**1132**	**1089**
**1139**	**1101**
**1140**	**1108**
**1148**	**1117**
1149	**1123**
**1161**	1126
**1164**	**1132**
**1169**	**1139**
**1171***	**1140**
**1171***	**1148**
1182	**1161**
**1187**	**1164**
**1213**	**1169**
**1241**	**1171***
**1252**	**1171***
**1270**	1171*

CH-Alpha® lot no.	
**L115/1031**	**L88/1031**
**Number of peaks: 103**	**Number of peaks: 113**
800	800
**921**	818
**955**	831
**958**	897
**960**	899
**976**	920
979	**921**
**997**	**956**
**1009**	**958**
**1014***	**960**
**1014***	**977**
**1024**	985
**1032***	**997**
**1032***	1008
**1048**	**1009**
**1050**	**1014***
**1066**	**1014***
1071	**1024**
**1080**	**1032***
1084	**1032***
**1085**	**1048***
**1089**	**1048***
**1106**	1049
1112	1050
1120	1056
**1123**	1064
**1154**	**1066**
**1159**	**1080**
1179	**1085**
1205	1088
**1223**	**1089**
**1225**	1090
1236	1093
**1239**	1096
1245	**1106**
**1297**	1107
1298	**1123**
**1305**	1139
1335	**1154**
**1352**	**1159**
1362	1206
**1370**	**1223**
**1380**	**1225**
**1398**	**1239**
**1419**	1255
1437	1268
**1452**	1280
**1455**	**1297**
1460	**1305**
**1477**	1336
**1478**	1340
**1484**	1340
**1492**	**1352**
**1534**	1354

Peptan® B 2000 lot no.	
**1048665**	**1266793/x**
**Number of peaks: 154**	**Number of peaks: 196**
802	**810**
**810**	**811**
**811**	814
**815**	**815**
**820**	**820**
**821**	**821**
**823**	**823**
828	**841**
**841**	853*
**853**	**853***
**854**	**854**
**861**	**861**
**871***	864
**871***	**871***
**879**	**871***
898	873
900	**879**
926	885
938	891
942	892
958	911
976	922
**982**	930
**999***	959
**999***	967
**1008**	971
**1021**	980
1026	**982**
1032	985
**1048**	993
**1049**	994
1050	**999***
**1064**	**999***
**1066**	1001
1073	**1008**
**1078**	1009
**1080**	1017
**1089**	**1021**
1093	1022
**1096**	1028
1101	1029
**1104**	1036
1106	**1047**
**1108**	**1049**
1117	1052
**1123**	**1064**
1129	**1066**
**1137**	1068
**1139**	1072
**1155**	1076
1156	**1078**
**1171**	1079
**1184**	**1080**
**1187**	1086*

**1304**	**1187**
**1347***	1188
**1347***	1204
1349	**1213**
1351	1223
**1357**	**1241**
**1361**	1250
1362	**1252**
**1375**	1265
**1380**	**1270**
**1390**	1284
**1403**	1300
**1406**	**1304**
1407	1305
**1414**	1306
**1423**	1329
**1430**	**1347***
1437	**1347***
**1439**	**1357**
**1447**	**1361**
**1455**	1362
**1463**	1364
1464	1372
**1478**	**1375**
1484	**1380**
**1490**	1385
**1527**	**1390**
**1548**	**1403**
**1561**	**1406**
**1565**	**1414**
1584	**1423**
**1590**	**1430**
**1608**	**1439**
1610	**1447**
**1612**	**1455**
**1616**	**1463**
1624	**1478**
**1628**	**1490**
**1633**	1498
**1642**	**1527**
**1644**	**1548**
**1648**	**1561**
**1649**	**1565**
**1656**	1569
1657	**1590**
1661	**1608**
**1664**	**1612**
**1673**	**1616**
1678	1620
**1689**	**1628**
1697	**1633**
1707	1635
**1715**	**1642**
1731	**1644**
1747	**1648**
**1759**	**1649**
**1761**	1651
**1770**	**1656**

**1545**	**1370**
**1550**	**1380**
1555	**1398**
1571*	1403
**1571***	**1419**
**1584**	**1452**
**1589**	**1455**
1645	1461
**1647**	1463
**1652**	1464
**1668**	**1477**
**1669**	**1478**
**1685**	1479
1687	**1484**
**1692**	**1492**
**1704**	1500
**1721**	1518
1726	1532
1831	**1534**
1833	**1545**
1844	**1550**
1859	**1571**
1886	**1584**
1904	**1589**
**1920**	1619
1948	1636
1957	**1647**
1961	**1652**
2019	1660
2037	**1668**
2039	**1669**
**2055**	**1685**
2265	**1692**
2343	**1704**
**2366***	1705
2366*	**1721**
**2382**	1732
**2398**	1759
**2465**	1762
**2481**	1913
2528	**1920**
2534	**2055**
**2538**	2113
**2564**	2199
**2750**	2343
**2789**	**2366**
2802	**2382**
3968	**2398**
3984	**2465**
	**2481**
	2534
	**2538**
	**2564**
	**2750**
	**2789**
	2797
	2803
	3773

1203	1086*
1225	**1089**
1238	1091
1253	1096*
**1276**	**1096***
**1281**	**1104**
1285	**1108**
1324	1113
**1325**	1116
**1335**	**1123**
1341	1124
**1344**	1128
**1350**	1133
1356	**1137***
**1357**	1137*
**1362**	**1139**
1366	1140
1371	1148
**1372**	1149
**1380**	**1155**
**1390**	1156
**1395**	1164
1397	1168
**1410**	1169
**1421**	**1171**
**1423**	1179
**1431**	**1184**
**1433**	**1187**
**1437**	1195
**1439**	1216
**1442**	1221
1455*	1224
**1455***	1241
**1458**	1250
1463	1258
1464	1272
**1467**	**1276**
1471	**1281**
**1484**	1305
1490	1322
1528	1324
**1533**	**1325**
1539	1329
1568	**1335**
1584	1340
**1605**	**1344**
**1628**	1347
**1633**	**1350**
**1635**	**1357**
**1644**	**1362**
1656	**1372**
**1664**	1374
**1692**	1375
1697	**1380**
**1726**	1385
1759	**1390**
**1760**	1392
**1761**	**1395**

1786	**1664**
**1844**	**1673**
**1860**	**1689**
1863	1702
**1873**	**1715**
**1874**	1721
**1889**	1743
**1891**	1745
1905*	**1759**
**1905***	1761*
**1911**	**1761***
1913	**1770**
1921	**1844**
1945	1853
**1962**	**1860**
**2013**	**1873**
2019	**1874**
**2050**	**1889**
**2107**	**1891**
**2147**	**1905**
**2193**	1908
**2265**	**1911**
**2281**	1929*
2366	1929*
**2372**	**1962**
**2379**	**2013**
**2451**	2025
**2475**	2030
2619	2043
2637	**2050**
**2742**	2066
2743	2082
2814	**2107**
2897	**2147**
	2161
	2175
	**2193**
	**2265**
	**2281**
	2346
	**2372**
	**2379**
	2391
	**2451**
	**2475**
	2590
	2636
	**2742**
	2753
	2874
	3127
	3312
	3331
	3347

3969

1784	1399
1800	1400
1811	**1410**
1841	**1421**
1844	**1423**
**1851**	**1431***
**1864**	1431*
**1866**	**1433**
**1877**	**1437**
1899	**1439**
**1905**	**1442**
1909	1443
**1911**	**1455**
1912	**1458**
1915	1465
1923	**1467**
**1939**	**1484**
**1957**	1491
**1973**	1500
**1976**	1515
2043	1518
2067	**1533**
2070	1536
2139	1562
**2161**	1565
**2175**	1568
2207	1576
2218	1589
2245	1590
2274	**1605**
2280	**1628**
**2296**	**1633**
2312	**1635**
2328	**1644**
2514	1650
2553	**1664**
2569	1689
2637	**1692**
2638	1702
2654	1705
2682	1713
2828	1715
	1721
	1722
	**1726**
	1738
	1755
	**1760**
	**1761**
	1785
	1794
	1801
	1831
	1847
	**1851**
	**1864**
	**1866**
	**1877**

1889
**1905**
**1911**
1916
**1939**
**1957**
**1973**
**1976**
2008
2010
2026
2044
2107
**2161**
2169
**2175**
2195
2238
2240
**2296**
2508
2514
2590
2637
2709
3026

Suppl. Table 2. Comparative analysis of MALDI-TOF mass spectra obtained from two Peptan® B 2000, two Mobiforte®, or two CH-alpha® batches. The peptides from each batch were separated into different fractions by reverse phase-HPLC according to their polar properties. The mass numbers of the peptides were then determined using MALDI-TOF-MS, and the identified peaks being common in all three replicate measurements are presented by their mass-to-charge ratio (*m/z*). Peptides found in both batches are written in bold, whereas those peptides with the same *m/z* but different fractions numbers or only a minimally deviating m/z are marked with *. The numbers of common peaks found in the two batches of Mobiforte®, CH-Alpha® and Peptan® B 2000 were 109 (= 54%), 63 (= 41%), and 83 (= 31%), respectively.

**CH-Alpha® lot no.**		
**L115/1031**	**L88/1031**	**L170/1031**
**Number of Peaks: 103**	**Number of Peaks: 113**	**Number of Peaks: 95**
800	800	818
**921**	818	821
**955**	831	841
958	897	891
960	899	898
**976**	920	913
979	**921**	920
**997**	**956**	**921**
**1009**	958	937
1014*	960	938
**1014***	977	**955**
**1024**	985	**976**
**1032***	**997**	985
1032*	1008	**997**
**1048**	**1009**	**1009**
**1050**	1014*	**1014**
**1066**	**1014***	**1024**
1071	**1024**	**1032**
**1080**	**1032***	**1048**
1084	1032*	1049
1085	**1048***	**1050**
**1089**	1048*	1064
**1106**	1049	**1066**
1112	**1050**	1078
1120	1056	**1080**
**1123**	1064	1084
**1154**	**1066**	1088
**1159**	**1080**	**1089**
1179	1085	1105
1205	1088	**1106**
**1223**	**1089**	1121
**1225**	1090	**1123**
1236	1093	1136
**1239**	1096	**1154**
1245	**1106**	**1159**
1297	1107	**1223**
1298	**1123**	**1225**
1305	1139	**1239**
1335	**1154**	1245
**1352**	**1159**	1255
1362	1206	1269
**1370**	**1223**	1285
**1380**	**1225**	1335
**1398**	**1239**	1336
**1419**	1255	1340
1437	1268	1350
**1452**	1280	**1352**
1455	1297	1356
1460	1305	**1370**
1477	1336	**1380**
**1478**	1340*	**1398**
1484	1340*	**1419**
**1492**	**1352**	**1452**
**1534**	1354	1461

**1545**	**1370**	1463
1550	**1380**	**1478**
1555	**1398**	**1492**
1571*	1403	**1534**
**1571***	**1419**	**1545**
1584	**1452**	**1571**
**1589**	1455	**1589**
1645	1461	1636
**1647**	1463	**1647**
**1652**	1464	**1652**
1668	1477	1660
**1669**	**1478**	**1669**
**1685**	1479	**1685**
1687	1484	1687
**1692**	**1492**	1688
**1704**	1500	**1692**
**1721**	1518	**1704**
1726	1532	**1721**
1831	**1534**	1732
1833	**1545**	1762
1844	1550	1803
1859	**1571**	1843
1886	1584	1868
1904	**1589**	1886
**1920**	1619	**1920**
1948	1636	1957
1957	**1647**	1977
1961	**1652**	2009
2019	1660	2019
2037	1668	2039
2039	**1669**	**2055**
**2055**	**1685**	2113
2265	**1692**	2264
2343	**1704**	2366*
**2366***	1705	**2366***
2366*	**1721**	**2382**
**2382**	1732	**2398**
**2398**	1759	**2538**
2465	1762	2770
2481	1913	2997
2528	**1920**	3517
2534	**2055**	
**2538**	2113	
2564	2199	
2750	2343	
2789	**2366**	
2802	**2382**	
3968	**2398**	
3984	2465	
	2481	
	2534	
	**2538**	
	2564	
	2750	
	2789	
	2797	
	2803	
	3773	

Suppl. Table 3. Comparative analysis of MALDI-TOF mass spectra obtained from three CH-alpha® batches. The peptides of each batch were separated into different fractions by reverse phase-HPLC according to their polar properties. The mass numbers of the peptides were then determined using MALDI-TOF-MS. The identified peaks being common in all three replicate measurements are presented by their mass-to-charge ratio (*m/z*). Peptides found in all three batches are written in bold, whereas those peptides with the same *m/z* but different fractions numbers or only a minimally deviating *m/z* are marked with *. The number of common peaks found in all three CH-Alpha® batches was 45 (=25%).
